# Subthreshold Depression and Clinically Significant Depression in an Italian Population of 70–74-Year-Olds: Prevalence and Association with Perceptions of Self

**DOI:** 10.1155/2017/3592359

**Published:** 2017-03-14

**Authors:** Roberta Vaccaro, Paola Borrelli, Simona Abbondanza, Annalisa Davin, Letizia Polito, Mauro Colombo, Silvia Francesca Vitali, Simona Villani, Antonio Guaita

**Affiliations:** ^1^“Golgi Cenci” Foundation, Corso San Martino 10, 20081 Abbiategrasso, Italy; ^2^“C. Golgi” Geriatric Institute, Piazza Samek 5, 20081 Abbiategrasso, Italy; ^3^Unit of Biostatistics and Clinical Epidemiology, Department of Public Health, Experimental and Forensic Medicine, University of Pavia, Via Forlanini 2, 27100 Pavia, Italy

## Abstract

Estimates of depressive disorders in the elderly vary depending on how cases are defined. We estimated the prevalence of subthreshold depression (SD) and clinically significant depression (D) in a population of 70–74-year-olds. We also looked for associations with sociodemographic factors and perceptions of self. Participants underwent a multidimensional assessment (social, medical, and neuropsychological). The estimated prevalence of SD was 15.71% (95% CI: 13.70–17.72), while that of D was 5.58% (95% CI: 4.31–6.85). Multinomial logistic regression analysis revealed that female gender and dissatisfaction with family relationships were related to SD and D. A self-perception of physical age as older than actual age (but not comorbidity) and greater self-perceived stress caused by negative life events both increased the probability of SD. The likelihood of D was decreased in those who perceived their own health as good, whereas a self-perception of mental age as older than actual age and dissatisfaction with relationships with friends were both significantly associated with D. Both SD and D emerged as key problems in our population. Female gender and self-perceptions of various characteristics, which can be explored through simple questions, are associated with late-life depression in elderly people independently of their actual physical condition and other characteristics.

## 1. Introduction

Depressive disorders in aging are associated with increased physical morbidity, lower functional status, and a higher risk of dementia, and they are a major economic and social burden on families and on society [1]. Late-life depression has been shown to occur along a spectrum ranging from subthreshold depression to clinically significant depression [2]. By definition, individuals with subthreshold depression have at least one key symptom of depression but insufficient other symptoms and/or functional impairment to meet the criteria for a full diagnosis [3]; nevertheless, their problems have a significant impact on their quality of life [2]. Clinically significant depression is a broad condition in which depressed mood and/or loss of pleasure in most activities are prominent features. Formal diagnoses of depressive disorders are usually made using the International Classification of Diseases or the Diagnostic and Statistical Manual of Mental Disorders (DSM) systems, and they are based on the severity of the disorder, the degree of functional impairment, and the duration of symptoms. However, this categorical approach could be difficult to apply in elderly people with comorbidities [4], in whom mood disorders can be masked by extensive somatic symptoms and drug treatments [5]. Moreover, elderly subjects tend to underreport depressed mood [6]. Instead of the categorical approach, some epidemiological studies have adopted a dimensional approach to this issue, using depressive symptoms questionnaires, that is, the Geriatric Depression Scale (GDS) and the Center for Epidemiological Studies Depression (CES-D) scale. As noted by Fiske and colleagues [5], studies that measure depressive symptoms through questionnaires report higher rates of depression than those adopting a categorical approach. Thus, international data on the prevalence of depressive symptoms in the elderly vary considerably, with reported rates ranging from 16% (referring to subthreshold depression) and 1% (referring to clinically significant depression) [1, 7]. In the present study, clinician-rated DSM diagnoses of depression were considered together with additional features (e.g., number of depressive symptoms reported on a depressive symptoms questionnaire) in order to obtain estimates of the rate of depressive conditions in this population.

Many social and clinical conditions, such as older age, female gender, low education, being single or uncoupled, living alone, physical illness, and low cognitive functions, in particular executive ones, are known to be associated with both subthreshold and clinically significant depression [5, 8]. On the other hand, primary lifetime occupation per se shows no relationship with depression in older people [9].

One aspect of depression in the elderly not yet studied in depth is the role of certain perceptions of self (across a range of variables) that may conceivably be associated with mood. Indeed, even though self-perceived health has been found to reflect subjective health-related quality of life in the elderly and appears to show a strong relationship with several health and social characteristics in this population, although, not with depressive symptoms [10], the relationship between self-perceived age and depressive disorders has not yet been studied. It may be of interest to do so, however, given that an increased risk of mortality has been reported in elderly people who felt older than their chronological age [11]. Social support is an important contributor to well-being, but the few studies that have investigated older individuals' satisfaction with their social relationships have given inconsistent results [12]. Negative life events are another contributor to greater depressive symptoms in older people [13], but there are no studies on the influence, on depression in the elderly, of perceived stress, or feelings, triggered by negative life events.

The primary aim of the present study was to estimate the prevalence rates of subthreshold depression and clinically significant depression in a section of the elderly Italian population (those aged 70–74 years) living in Abbiategrasso, a northern Italian town on the outskirts of Milan. The secondary aim was to evaluate social, medical, cognitive, and self-perception variables in this population and the influence of these factors on the presence of subthreshold depression and clinically significant depression.

## 2. Materials and Methods

### 2.1. Participants

The participants were drawn from the 1321 responders in the cross-sectional phase of InveCe.Ab (Invecchiamento Cerebrale in Abbiategrasso,* Brain Aging in Abbiategrasso*), an ongoing longitudinal population-based study of brain aging and dementia [14], which began on November 1, 2009 (ClinicalTrials.gov, NCT01345110). InveCe.Ab had a response rate of 80.4%, and no differences in age, birth area, or level of education were found between the responders and the nonresponders [15]. For the purposes of the present study, we excluded individuals who refused medical or neuropsychological assessment and therefore had no definitive diagnosis (*n* = 15) and those with a diagnosis of dementia (*n* = 39), psychosis (*n* = 11), or mental retardation (*n* = 2). Thus, the statistical analysis was performed on the data referring to the remaining 1254 subjects.

All the study procedures were in accordance with the Declaration of Helsinki and the study protocol was approved by the Ethics Committee of the University of Pavia on October 6, 2009 (Committee report 3/2009). In summary, InveCe.Ab is a single-step, multidimensional study, in which all the participants underwent the same assessment (social, medical, and neuropsychological), performed by trained geriatricians and neuropsychologists. All the participants gave their written informed consent to the use of their personal data.

### 2.2. Variables

#### 2.2.1. Subthreshold Depression and Clinically Significant Depression

The primary endpoint was depressive status classified as subthreshold depression (SD), clinically significant depression (D), or without depression (noD). The presence of depressed mood was evaluated as part of the medical and neuropsychological assessment. Following the guidelines of the National Institute of Clinical Excellence (NCCMH 2010), SD was defined as the presence of at least one key symptom of depression but with insufficient other symptoms to meet the criteria for a diagnosis of depression. In accordance with the DSM-IV-TR [16] system, at least five symptoms out of nine had to be present for a diagnosis of major depression, and they had to have been present for at least two weeks. A diagnosis of dysthymia was instead based on the presence of an at least two-year history of depressive mood plus at least two out of six symptoms. In our study, both conditions were considered to constitute D. Many elderly people receive drug treatments and present interfering pathologies, and when these coexist with depressive symptoms they constitute, according to the DSM-IV-TR, an exclusion criterion for a diagnosis of depression. In order to overcome this limitation and strengthen the case definitions used, we considered several additional features. Accordingly, SD could be deemed present if the patient showed a clinical impression of depression plus one or two of the following additional features: (i) a positive history of depression, (ii) use of antidepressant or anxiolytic/hypnotic drugs, (iii) a score of ≥6 on the 15-item Geriatric Depression Scale (GDS-15; [17]), and (iv) positive responses to two key questions on depressed mood, taken from the CES-D [18]: “During the past month, have you often been bothered by feeling down, depressed, or hopeless?” and “during the past month, have you often been bothered by little interest or pleasure in doing things?” D was also taken to correspond to the presence of a clinical impression of depression plus at least three of the above-mentioned features (in this case, applying a GDS-15 threshold score of ≥8).

All individuals not meeting the above-mentioned criteria for SD or D were considered to be without depression (noD).

#### 2.2.2. Sociodemographic Characteristics

The sociodemographic characteristics considered in the present survey were age (70–75), gender (female and male), years of education (≤5 or >5), marital status (defined as coupled [currently married/cohabiting] or single [never married]/uncoupled [separated/divorced/widowed]), living situation (living with spouse and/or others versus living alone), and primary lifetime occupation (considering a series of categories. housewife, blue collar worker, and white collar worker, adapted from the nine classes established by Italian National Institute of Statistics) [19].

#### 2.2.3. Comorbidity Index

The participants' general health was evaluated using the Cumulative Illness Rating Scale. This is a standardized tool for detecting multimorbidity, in which a five-level Likert scale is used to rate the severity of medical illness for each of 14 items referring to different domains [20], as follows: 1, no impairment to the organ/system; 2, mild impairment; 3, moderate impairment; 4, severe impairment; 5, extremely severe impairment. In this way, it is possible to calculate a comorbidity index that represents (after excluding the item Psychiatric/Behavioural diseases) the total number of domains in which moderate or severe and extremely severe levels of pathology are reported.

#### 2.2.4. Cognition

As part of the neuropsychological assessment global cognition was evaluated using the Mini-Mental State Examination (MMSE), whose items investigate orientation, registration and recall, attention, language, following commands, and figure copying [21], executive functions were evaluated using the free-hand Clock Drawing Test (CDT) [22].

#### 2.2.5. Perceptions of Self

Perceptions of self were investigated using various self-perception indices. During the social assessment, the subjects were asked to rate their overall health as poor, fair, or good. Self-perception of age was assessed considering two subindices: self-perceived mental and self-perceived physical age. Self-perceived mental age was determined by asking the patient “Do you feel mentally younger, the same as, or older than your actual age?” while the question concerning self-perceived physical age was “Do you feel physically younger, the same as, or older than your actual age?” The answers to these questions were classified as younger than actual age, equal to actual age, or older than actual age [11]. Satisfaction with relationships with family and friends was classified as satisfied or not satisfied. We also investigated negative life events, using the Geriatric Adverse Life Events Scale, a 26-item self-report checklist investigating the occurrence of such events over the previous 12 months. The participants were required to indicate whether each adverse life event they reported had caused them stress (rating it on a three-point Likert scale: 1, not at all stressful; 2, somewhat stressful; 3, very stressful) or affected their mood (in this case, they indicated the impact of the event using a five-point Likert scale, indicating whether it had left them feeling 1. much better; 2, better; 3, the same; 4, worse; 5, much worse). In addition to the number of negative events score, each patient was assigned two global scores, calculated by summing all their Likert scale scores for stress (stress score) and feelings (feeling score), respectively [13].

### 2.3. Statistical Analysis

Mean values with standard deviation (sd), or medians with interquartile range (iqr) if data lacked normality, were used to summarize the quantitative variables, while percentages were used to describe the categorical variables. To evaluate associations between depressive status (SD, D or noD) and categorical variables, the chi-square test or Fisher's exact test was performed. Similarly, to evaluate relationships with quantitative factors, a parametric analysis of variance (ANOVA) or corresponding nonparametric test (Kruskal-Wallis rank test) was applied. If the associations with different depressive status were significant, then multiple comparison tests with Bonferroni's correction were applied taking noD as the reference status. Normality distribution was assessed using the Shapiro-Wilk test. A multinomial logistic regression model was performed to investigate the association of depressive status with several social, medical, cognitive, and self-perception variables using noD as the reference category in the model. The results were reported as the relative risk ratios (RRRs) with 95% confidence intervals (95% CI). Multinomial logistic regression allowed simultaneous comparison of the three categories of depressive status while adjusting for all other variables in the model. The goodness-of-fit of the multinomial model was assessed by means of the Hosmer-Lemeshow test. A *p* value of less than 0.05 was significant, except for multiple comparisons (*p* value/number of comparisons equal to 2). In any case, the *p* value of multiple comparisons was reported to the threshold 0.05. All analyses were conducted using STATA/SE for Windows, version 12 (StataCorp 2012. Stata Statistical Software: Release 12. College Station, TX: StataCorp LP).

## 3. Results

The prevalence of SD among the 1254 elderly subjects was 15.71% (95% CI: 13.70–17.72), while that of D was 5.58% (95% CI: 4.31–6.85).

Depressive status was differently associated with all the demographic characteristics except for years of education (*p* = 0.266). In particular, the D subjects were slightly older than the noD ones (*p* = 0.047). In addition, individuals with SD or D were more likely to be women (*p* < 0.001), single/uncoupled (*p* < 0.001), living alone (*p* < 0.001), and housewives (*p* < 0.001) than noD subjects (Table 1). Similarly, comorbidity, cognitive, and self-perception variables showed statistically significant differences between the three categories of depressive status (Table 2). Of note, the D individuals showed worse global cognition (MMSE total score) and poorer executive functions (CDT total score) than the noD group (*p* = 0.007 and *p* = 0.008, resp.). Furthermore, the D and SD subjects, compared with the noD subjects, had experienced more negative life events and recorded higher stress and feeling scores (*p* < 0.001 and *p* < 0.001, resp.).

### 3.1. Variables Associated with Subthreshold Depression and Clinically Significant Depression: Multinomial Logistic Regression Analysis

According to the results of the multinomial logistic regression analysis (Table 3), the variables associated with both SD and D, independently of other factors, were gender (*p* < 0.001) and satisfaction with relationships with family (SD, *p* < 0.05; D, *p* < 0.001).

Specifically, in females with respect to males, the relative risk of having SD versus noD was increased almost threefold, while the relative risk of having D as opposed to noD was increased fivefold.

In other words, females were more likely to belong to SD or D group versus noD than males. Similarly, subjects dissatisfied with their relationships with their family compared with those who declared themselves satisfied were more likely to have SD (one and a half times) or D (around three times) as opposed to noD status.

Conversely, variables significantly associated only with SD, independently of other factors, were self-perception of physical age (*p* < 0.001) and stress score (*p* < 0.01). In particular, subjects who perceived their own physical age as older than their actual age with respect to those who perceived their own physical age as equal to their actual age were more likely (three times) to have SD as opposed to noD (3 times), while those with a higher stress score were more likely (1.2 times) to have SD rather than noD.

Instead, the variables significantly associated with D, independently of other factors, were self-perception of health (*p* < 0.01), self-perception of mental age (*p* < 0.001), and satisfaction with relationships with friends (*p* < 0.001) (Table 3). Subjects with a positive perception of their health status were less likely to have D versus noD than those perceiving their health as poor (showing an almost 87% decrease in the RRR of having D as opposed to noD). Instead, subjects who perceived their mental age as older than their actual age with respect to those who perceived their mental age as equal to their actual age were more likely (around 4 times) to have D rather than noD. Finally, subjects who were not satisfied with their relationships with friends were more likely to belong to D group versus noD than those who were satisfied (almost twofold).

No other variable was associated with SD or D. The Hosmer-Lemeshow goodness-of-fit test indicated that the model described the data well (chi-square (8) = 11.36, *p* = 0.79).

## 4. Discussion

We conducted a comprehensive investigation of late-life depression in which additional features were considered together with clinician-rated DSM diagnoses of depression. The study showed prevalence rates of 15.71% for SD and 5.58% for D in our elderly population. This result confirms that SD is a problem not only in young people and adults but also in the elderly, in line with data reported in other, similar studies [8]. It is possible that the lower estimated rates reported elsewhere [7] may, as already suggested, be due to other authors' use of nosological classifications alone.

Furthermore, our data indicate that clinicians should carefully monitor elderly subjects not only for D but also for SD, given the high risk of this latter condition evolving into more severe depression [23]. Moreover, both these conditions need to be identified so that targeted interventions can be prescribed. Recent literature evidence suggests that problem-solving therapy and cognitive behavioural therapy may be feasible and effective options for elderly people with depressive symptoms [24]. Physical exercise, whether performed at a low (yoga or similar), moderate, or vigorous intensity (aerobic training), has also been shown to be effective in treating mild-to-moderate depression [25].

### 4.1. Variables Associated with Subthreshold Depression and Clinically Significant Depression

In accordance with previously reported findings [5, 8], univariate analysis of our data showed that SD and D were significantly associated with older age, female gender, being single or uncoupled, and living alone. Low education was not found to be associated with depressive status, in accordance with data from SHARE [26]. This finding may reflect that fact that the majority of the InveCe.Ab population had not been educated beyond primary school level. We also found a significantly higher percentage of housewives among the SD and D subjects, which supports the idea that having less opportunity to interact with others is a negative circumstance [9]. In our study, the SD and D subjects had poorer global cognition (MMSE total score) and executive functions (CDT total score) than those without depression, although the multinomial regression analysis revealed no significant association with depressive status. This result is in line with a previous report in which depressive symptoms in older people were not found to be associated with cognitive functions [27].

### 4.2. Variables Associated with Subthreshold Depression and Clinically Significant Depression: Multinomial Logistic Regression Analysis

The multinomial regression analysis identified three groups of variables showing associations with depressive status: those associated with both SD and D, those associated only with SD, and those associated only with D, independently of comorbidity and other characteristics.

With regard to the first group, females were more likely to be classified as SD or D versus noD than males, confirming that the female gender is more prone to depressive symptoms [5]. Furthermore, dissatisfaction, as opposed to satisfaction, with family relationships was found to increase a subject's probability of being classified as SD or D as opposed to noD, a finding in line with recent data on the role of poor quality of social relationships in depressive conditions [28]. These two factors were consistently associated with depression, that is, across the SD and D groups, supporting the idea of a continuum, as previously reported [2].

With regard to the second group, having an older perceived than actual physical age, as opposed to a perception consistent with actual age, increased the probability of being classified as SD versus noD. This, together with the absence of relationships between comorbidity and SD or D in this sample, suggests that subjective but not objective physical difficulties are implicated in SD. It is well known that physical comorbidity is related to a higher level of depression [29], but there are no studies that have evaluated the effect of subjective perceptions in addition to actual physical problems. Furthermore, in our study, subjects with a higher level of perceived stress caused by negative life events were more likely to be classified as SD, but not D, rather than noD. These data conflict with the results described by Devanand and colleagues [13]. A possible explanation for this discrepancy is that the impact of negative life events on depression may depend on whether or not the event is still ongoing [30]. However, in our study, we did not collect this information; thus further investigations are needed.

As regards the third group of variables showing associations with depressive status, subjects who considered their health as good as opposed to poor were less likely to be classified as D versus noD, a result in line with the findings of others [31]. Subjects perceiving their own mental age as older than their actual age were instead more likely to belong to the D versus the noD group than those with an accurate perception of their mental age, a finding which supports the idea that depressed subjects have a negative perception of their own mental effectiveness. To our knowledge, no previous study has evaluated self-perceived age, physical or mental, in relation to depressive conditions. Finally, subjects dissatisfied with their relationships with friends were more likely to be classified as D versus noD than satisfied ones, a finding which supports the idea that the quality of social relationships plays a role in determining not only subjective well-being but also depressive conditions [28]. Taken together, these results support the importance of perceptions of self in depressive status.

The results of our study show that SD and D are key problems in the elderly and that they are associated with mood independently of actual physical conditions and other characteristics; it also showed that they are subjective dimensions that can be easily explored through simple questions. Perceptions of self may offer the clinician an individualised profile of the patient with SD or D.

The strengths of the present study include the fact that it is a population study with a good response rate [14], in which all the participants underwent a comprehensive medical and neuropsychological assessment; furthermore, a multidimensional approach was used in order to estimate the presence of depressive disorders. In this context, self-perceptions, specifically of health status, mental and physical age, satisfaction with relationships, and stress due to negative life events emerged as the most informative variables. However, we acknowledge that our study has some limitations: (i) its cross-sectional design allowed us to investigate the strength but not the directionality of associations (since the InveCe.Ab study is an ongoing prospective study, this aspect may be addressed in a subsequent work); (ii) we could not assess the presence of depressive disorders in the nonresponders (that said, there were no differences in age, birth area, or level of education between the responders and the nonresponders), and (iii) positive life events, chronic stressors, and age at onset of depressive disorders were not assessed.

## 5. Conclusion

Through a comprehensive investigation of depressive conditions, we obtained estimates of the rates of SD and D in a sizeable sample of elderly subjects (70–74-year-olds). The results of our study confirmed that both SD and D are key problems in the elderly. They also confirmed that female gender and self-perceptions of various variables (health status, mental and physical age, satisfaction with relationships, and stress linked to negative life events) are associated with late-life depression, independently of physical comorbidity and other factors.

## Figures and Tables

**Table 1:** Sociodemographic characteristics in elderly subjects with subthreshold depression (SD), with clinically significant depression (D), and without depression (noD).

	SD (*n* = 197)	D (*n* = 70)	noD (*n* = 987)	*p* value^†^
*Age*, mean ± sd (years)	73 ± 1.39	73.2 ± 1.29	72.7 ± 1.41	0.013
*Gender*, *n* (%)				<0.0001
Male	52 (26%)	11 (19%)	519 (52%)	
Female	145 (74%)	59 (81%)	468 (48%)	
*Education*, *n* (%)				0.266
≤5 years	122 (62%)	42 (60%)	545 (55%)	
>5 years	75 (38%)	28 (40%)	442 (45%)	
*Marital status*, *n* (%)				<0.0001
Coupled	106 (54%)	36 (51%)	706 (71%)	
Single/uncoupled	91 (46%)	34 (49%)	281 (29%)	
*Living situation*, *n* (%)				<0.0001
Living with spouse and/or others	124 (63%)	41 (59%)	761 (77%)	
Living alone	73 (37%)	29 (41%)	226 (23%)	
*Primary lifetime occupation*, *n* (%)				<0.0001
Housewife	34 (17%)	25 (36%)	124 (13%)	
Blue collar worker	95 (48%)	28 (40%)	504 (51%)	
White collar worker	68 (35%)	17 (24%)	359 (36%)	

^†^ *p* values are for *F* or *Kruskal-Wallis rank test* or *Pearson's chi-square* test.

**Table 2:** Comorbidity, neuropsychological, and self-perception variables in elderly subjects with subthreshold depression (SD), with clinically significant depression (D), and without depression (noD).

	SD (*n* = 197)	D (*n* = 69)	noD (*n* = 985)	*p* value^‡^
*Comorbidity Index (range 0–13)*, median (iqr^†^)	2 (3–1)	3 (4–2)	2 (3–1)	0.0001
*MMSE total score (range 0–30)*, median (iqr^†^)	28 (29–27)	27 (29–26)	29 (29–27)	0.002
*CDT total score (range 0–20)*, mean ± sd	18 ± 1.92	18 ± 1.79	19 ± 1.77	<0.0001
*Self-perception of health status*, *n* (%)				<0.0001
Poor	3 (2%)	5 (7%)	6 (1%)	
Fair	101 (51%)	46 (67%)	265 (27%)	
Good	93 (47%)	18 (26%)	714 (72%)	
*Self-perception of mental age*, *n* (%)				<0.0001
Equal to actual age	88 (45%)	19 (28%)	547 (56%)	
Younger than actual age	80 (41%)	36 (53%)	395 (40%)	
Older than actual age	29 (15%)	13 (19%)	37 (4%)	
*Self-perception of physical age*, *n* (%)				<0.0001
Equal to actual age	60 (31%)	12 (18%)	482 (49%)	
Younger than actual age	90 (46%)	33 (49%)	444 (45%)	
Older than actual age	45 (23%)	22 (33%)	53 (6%)	
*Satisfaction with relationships with family*, *n* (%)				<0.0001
Satisfied	180 (92%)	59 (83%)	996 (98%)	
Not satisfied	15 (8%)	12 (17%)	18 (2%)	
*Satisfaction with relationships with friends*, *n* (%)				<0.0001
Satisfied	164 (87%)	42 (65%)	885 (91%)	
Not satisfied	25 (13%)	23 (35%)	84 (9%)	
*Number of negative life events*, mean ± sd	2.44 ± 1.40	2.42 ± 1.33	1.85 ± 1.29	<0.0001
*Stress score (range 1–3)*, median (iqr^†^)	5 (7–3)	5 (8–3)	3 (5–2)	0.0001
*Feeling score (range 1–5)*, median (iqr^†^)	8 (12–5)	9 (13–5)	6 (9–3)	0.0001

^†^iqr = interquartile range.

^‡^ *p* values are for *F* or *Kruskal-Wallis rank test *or* Pearson's chi-square* test.

**Table 3:** Multinomial logistic regression for identifying factors associated with depressive status (reference group: noD).

	SD versus noD	D versus noD
	RRR (95% CI)	RRR (95% CI)
*Age*, years	1.08 (0.95–1.24)	1.08 (0.87–1.35)
*Gender*		
Male	1	1
Female	2.69^*∗∗∗*^ (1.75–4.16)	4.78^*∗∗∗*^ (1.99–11.50)
*Education*, years		
≤5 years	1	1
>5 years	0.94 (0.59–1.49)	2.12 (1.00–4.49)
*Marital status*		
Coupled	1	1
Single/uncoupled	1.11 (0.83–1.50)	0.97 (0.59–1.57)
*Living situation*		
Living with spouse and/or others	1	1
Living alone	1.13 (0.92–1.39)	1.17 (0.84–1.63)
*Primary lifetime occupation*		
Blue collar worker	1	1
Housewife	0.82 (0.48–1.40)	1.18 (0.54–2.55)
White collar worker	1.29 (0.80–2.08)	0.71 (0.30–1.70)
*Comorbidity Index*	1.02 (0.90–1.16)	1.02 (0.83–1.25)
*MMSE total score *	0.96 (0.88–1.05)	0.92 (0.81–1.06)
*CDT total score*	0.97 (0.87–1.08)	0.89 (0.77–1.04)
*Self-perception of health status*		
Poor	1	1
Fair	1.73 (0.28–10.4)	0.40 (0.77–2.08)
Good	0.99 (0.16–6.08)	0.13^*∗∗*^ (0.02–0.75)
*Self-perception of mental age*		
Equal to actual age	1	1
Younger than actual age	0.81 (0.52–1.25)	1.56 (0.72–3.38)
Older than actual age	1.93 (0.90–4.15)	4.26^*∗∗*^ (1.42–12.76)
*Self-perception of physical age*		
Equal to actual age	1	1
Younger than actual age	1.38 (0.87–2.17)	1.44 (0.61–3.38)
Older than actual age	3.06^*∗∗∗*^ (1.54–6.08)	2.14 (0.70–6.52)
*Satisfaction with relationships with family*		
Satisfied	1	1
Not satisfied	1.67^*∗*^ (1.09–2.56)	2.78^*∗∗∗*^ (1.70–4.53)
*Satisfaction with relationships with friends*		
Satisfied	1	1
Not satisfied	1.04 (0.78–1.39)	1.81^*∗∗∗*^ (1.28–2.56)
*Stress score*	1.21^*∗∗*^ (1.05–1.39)	1.12 (0.88–1.43)
*Feeling score*	0.97 (0.90–1.05)	1.00 (0.87–1.15)

^*∗*^ *p* value ≤ 0.05, ^*∗∗*^*p* ≤ 0.01, ^*∗∗∗*^*p* ≤ 0.001.
